# 

**Published:** 1879-05

**Authors:** 


### ILLINOIS STATE DENTAL SOCIETY.

The fifteenth annual meeting of the Illinois State Dental Society will be held in Springfield, Ill., commencing on Tuesday, May 13, 1879, at ten o'clock, a. m.

SUBJECTS FOR DISCUSSION.

1. President's Address.
2. What is Progression in Dentistry? M. H. Patten.
3. Pulp Extirpation and Root Filling. L. C. Ingersol.
4. Meckel's Cartilage—its Origin and Transitory Functions. M. S. Dean.
5. Dental Education. K. B. Davis.
6. Mechanical Dentistry. E. D. Swain.
7. Anæsthetics. A. W. Freeman.
8. Operative Dentistry. E. H. Stewart.
9. Action of Therapeutic Agents. A. E. Gibbs.

The Executive Committee, in the discharge of the duties devolving upon them in procuring a place, and in making arrangements for the approaching meeting, feel that nothing has been left undone that would in any way add to the interest of all concerned. And, feeling thus, they are now prepared to extend an earnest and cordial invitation to every dentist in Illinois and elsewhere to meet them this year at Springfield. Let every dentist receiving this circular consider himself specially invited. You will find warm hearts and welcome hands to greet you.

Clinics will form a very interesting as well as important feature at this meeting. Arrangements have been made for them, in both the operative and mechanical departments, by first-class operators. The demonstration in mechanical dentistry, by Dr. E. D. Swain, will be practical, from the impression to placing a gold plate in the mouth, and will, it is hoped, be witnessed by a large number of dentists.

Hotel accommodations are all that can be desired. Leland Hotel, per day, two dollars, two dollars and fifty cents and three dollars, according to location of rooms. St. Nicholas, per day, one dollar and fifty cents. The latter is recommended as a first-class hotel.

### *THE DENTAL REGISTER,*

A MONTHLY MAGAZINE DEVOTED TO THE INTERESTS OF

### THE DENTAL PROFESSION.

*Terms Reduced to $2 50 per Year. Single Copies 25c.*

*EDITED BY PROF. J. TAFT, D. D. S.,*

AND PUBLISHED BY

SPENCER & CROCKER, Ohio Dental Depot.

The volume commences in January and closes in December.

The REGISTER being issued monthly is always fresh, therefore Indispensable to the practitioner who desires to keep posted up with the new inventions and improvements which are daily developed. Neither expense nor effort will be spared to give value and variety to its contents. Articles will be illustrated with well executed engravings whenever they will add to the interest or assist to explanation.

Its extensive circulation and frequency of issue make it one of the BEST DENTAL ADVERTISING MEDIUMS in the United States.

All subscrptions must begin with January or July.

Local or special notices, five lines of less, will be inserted for $3.00 each insertion; over five lines, 50 cts. per line.

All advertising bills payable in advance, or at furthest, after the issue of the first number containing the advertisement. All transient advertisements to be paid for in advance.

☞CONTRIBUTIONS SOLICITED.

Exclusively Editorial Communications should be addressed to Editor.

The latest number of the REGISTER may be ordered with or without other goods at any time, and all letters should be addressed to

**SPENCER & CROCKER, Ohio Dental Depot, Cincinnati, O.**

